# Development of a short scale for assessing economic environmental aspects in patients with spinal diseases using Rasch analysis

**DOI:** 10.1186/s12955-017-0767-9

**Published:** 2017-10-10

**Authors:** Judith Gecht, Verena Mainz, Maren Boecker, Hans Clusmann, Matthias Florian Geiger, Markus Tingart, Valentin Quack, Siegfried Gauggel, Allen W. Heinemann, Christian-Andreas Müller

**Affiliations:** 1 Department of Medical Psychology and Medical Sociology, RWTH Aachen University, Pauwelsstr. 19, 52074 Aachen, Germany; 2 Department of Neurosurgery, RWTH Aachen University, Aachen, Germany; 3 Department of Orthopedic Surgery, RWTH Aachen University, Aachen, Germany; 4 Department of Physical Medicine and Rehabilitation, Feinberg School of Medicine, Northwestern University, Chicago, IL USA; 5 Center for Rehabilitation Outcomes Research, Rehabilitation Institute of Chicago, Chicago, IL USA

**Keywords:** Ecological environmental factors, ICF, Context factors, Rasch analysis, Scale development, Spinal diseases

## Abstract

**Background:**

Economic environmental factors represent important barriers to participation and have deleterious effects on quality of life (QOL) in persons with spinal diseases (SpD). While economic factors are anchored in the International Classification of Functioning, Disability and Health, their influence on QOL and participation from patients’ perspectives is an infrequent focus of research. The aim of the present research is to calibrate a culturally adapted Rasch-based questionnaire assessing economic QOL in patients with SpD.

**Methods:**

The 11-items of the German economic-QOL-scale were answered by 325 patients with SpD on a four-point Likert-scale. Fit to the Rasch measurement model was investigated by testing for stochastic ordering of the items, unidimensionality, local independence, and differential item functioning (DIF).

**Results:**

After adjusting for local dependency, fit to the Rasch model was achieved with a non-significant item-trait interaction (chi-square_df = 20_ = 34.8, *p* = 0.021). The person separation reliability equaled 0.88, the scale was free from age- or gender-related DIF, and unidimensionality could be verified.

**Conclusions:**

The Rasch-based German version of the economic-QOL-scale represents a suitable instrument to investigate the influences of economic factors on patients’ QOL at a group and individual level. It can be easily applied in research and practice and may be administered quickly in combination with other instruments. The short test duration implies a low test burden for patients and a minimum of time expenditure by clinicians when evaluating the results.

## Background

People with spinal diseases (SpD) face limitations in their everyday lives including limitations in functioning of activities in daily living, social as well as vocational participation, and important psychological consequences like major depression [1–8]. In Germany, the 12-months prevalence of dorsopathies, defined as back and neck pain that is present most part of the day and during a period of at least three months, was nearly 20.7% in 2009 [9] and the costs of illness amounted to 9 billion euros in 2008 [10]. Estimates of lifetime prevalence of back pain vary between 74% and 85% [11].

A framework to describe the aspects and consequences of disability is presented in the *International Classification of Functioning, Disability and Health* (ICF; [12]). The ICF reflects a biopsychosocial model of functioning and, thereby, provides a coherent view of different perspectives of health addressing the whole, complex notion of disability. In the ICF, the interactions between health conditions and contextual factors, i.e. environmental and personal influences on a person’s health status, influence disability and functioning. Functioning itself consists of the three aspects functioning at the level of body structures, functioning regarding activities by individuals themselves, and functioning in a social context. Disability therefore involves dysfunctioning at one or more of these levels resulting in impairments, activity limitations and/or participation restrictions, thereby referring to the negative aspects of the interaction between a person’s health condition and that person’s contextual factors. The ICF emphasizes that physical, social, political, and economic aspects of the environment are important precursors of health and may restrict opportunities for participation. The overarching role of participation is identified as a primary goal within the Standard Rules on the Equalization of Opportunities for Persons with Disabilities of the General Assembly of the United Nations [13, 14]. Following this, a key goal of rehabilitation, health care, and social services for people with SpD is to promote participation in everyday activities while taking into account the moderating effect of environmental influences. These different environmental factors often intersect and have cumulative negative influences on participation [4–7, 15, 16].

Among other environmental features, i.e., the built and natural environment, the application and availability of assistive technology, access to information and technology, system, services, and policies, transportation services and access, as well as social support and societal attitudes, economic environmental factors were consistently identified as having a critical influence on participation [5, 6]. Economic environmental factors may act as powerful facilitators to community participation and leisure activities but have also been identified as important barriers [5, 8, 17]. Individuals who experience insufficient economic resources, despite objective indicators to the contrary, e. g., an objectively sufficient salary level, can experience economic hardship, which in turn has a deleterious effect on quality of life (QOL) and participation in persons with SpD [17, 18]. Hammel and colleagues [5] asked community-dwelling people with spinal cord injury (SCI), stroke, and traumatic brain injury (TBI) to describe how participation was influenced by the adequacy of economic resources to live and participate in the community across a variety of environmental contexts and societal economic issues. Participants identified more distant economic influences, e.g., economic recessions and cutbacks, system reallocation of subsidies, and changes in benefits of policies governing resource allocation. More specifically, they referred to system-level economic issues, like inadequate subsidized income supports or lack of incentives in systems to return to work. Participants also identified specific economic issues in their proximal environment that influenced their QOL and participation, e.g., having sufficient income to afford market rent in ones’ community, costs for medication and other medical expenses, informal and nursing home care or services, accessible housing, assistive technology/equipment, home and vehicle modifications, transportation for work or leisure as well as having resources to dine out and socialize in one’s community [5, 8]. Compounding the costs of disability is the inability to return to one’s previous occupation or employment status. For instance, chronic unemployment or underemployment and associated income loss are significant barriers to QOL and participation [8].

Economic factors are anchored in the ICF (ICF taxonomy code e1650) and the evidence cited above highlights the detrimental effects of economic environmental aspects on QOL and participation in persons with SpD. However, economic environmental factors as *subjectively perceived* by the patient are not commonly conceptualized and, still, the influence of economic factors on QOL and participation is an infrequent focus of research [8]. This is surprising giving the enormous costs of disability, not only for the community but also for patients themselves. The scarcity of research on perceived economic QOL can partly be attributed to shortcomings of available instruments [8, 19, 20]. The concept of economic QOL reflects a distinctive construct that only few instruments measure [8]. Most of the available scales assessing economic aspects of QOL show psychometric deficiencies, are limited in scope, do not consider financial barriers related to disability, or the costs of health care and personal assistance. Economic factors receive hardly any consideration in QOL measures designed for disability populations. Other instruments focus solely on objective measures of financial status. Therefore, the available instruments do not reflect all of the relevant economic factors and financial needs of individuals with disabilities [8, 21]. Because an individual’s perception of an economic barrier to participation in life activities defines that person’s barrier to participation [8, 21], it is essential to investigate perceptions of the influence economic aspects on QOL by people with disabilities.

In order to fill this gap, Tulsky and colleagues [8] developed and evaluated a Rasch-based item bank and an instrument [22] measuring economic QOL among individuals with SCI, TBI, and stroke. Economic QOL is assessed by describing the economic barriers and facilitators of community participation and items focus on how financial resources influence satisfaction with one’s living situation, adequate and affordable health services, adequate and affordable food, affordable community recreational activities, and family and friend financial assistance.

The aim of the present research is the cultural adaptation and calibration of a Rasch-based short scale that can be applied to assess the relationship between economic QOL and participation in Germany and other German speaking countries. Therefore, a first necessary step is the construction of a psychometrically validated instrument in the German language that is adapted to the specificities of the German social system. To this end the items originally generated by Tulsky and colleagues [8, 22] are used as the basic item pool. In contrast to the original instrument [22], in the present German scale items distinguishing between basic economic needs and items that express more wealth or luxury are also included. Furthermore, the German scale comprises items accounting for cross-cultural differences as well as differences in the social and health systems between the USA and Germany. The resulting short scale shall be applied in medical and rehabilitation settings to identify economic barriers as well as possible resources in patients’ environments. Accordingly, interventions to promote participation and QOL can be adapted more appropriately to the subjectively experienced economic consequences of disability. Furthermore, rehabilitation processes can be initiated that are tailored more precisely to individuals’ living situations.

## Methods

### Study setting and patients

Data for the present study were collected as part of a broader project from in- and out-patients treated for SpD in a German neurosurgery and orthopedic surgery clinic between May 2015 and June 2016. Patients filled out the study material during waiting time in the hospital. In case the patients could not finish the questionnaires during the time in hospital they were given the opportunity to finish the material at home and return it within the next few days. Patients were eligible when they had a diagnosis of SpD according to the 10th revision of the International Classification of Diseases (ICD-10; [23]). Excluded were patients younger than 18 years of age, people with severe cognitive impairments and those deemed legally incompetent. Cognitive impairment was either self-reported (i.e. existence of a diagnosis beforehand) or was observed by the attending physician. During the study period in total 205 in- and 1152 out-patients with spinal diseases were approached for the study. At the day of consultation 327 patients refused participation for various reasons (e. g. “no interest”, “do not feel like it”, “too exhausting”, “pain” and “being too short before the operation”). Another 361 patients could not participate in the study because they did not meet inclusion criteria (e. g. “bad general health condition”, “having other diagnosis than spinal cord injury”, “severe cognitive impairment”). 76 patients had not sufficient knowledge of the German language to understand the questionnaires. Of the 587 study participants 6 patients canceled participation during the investigation in hospital and 262 patients did not send back the study material. Finally, a total of 325 patients (99.7% Caucasian) with a mean age of 55.1 ± 14.6 years completed the item pool. The characteristics of the calibration sample are shown in Table 1.

**Table 1:** Demographic characteristics of the calibration sample

Variable		Total Sample (*N* = 325)
Age^a^	<54.9^b^	162
	≥54.9^b^	163
Gender	female	199
	male	126
Type of admittance	out-patients	240
	in-patients	85
Diagnosis	lumbal stenosis	90
	lumbal disc herniation	57
	cervical disc herniation	22
	cervical myelopathy	22
	cervical and lumbal	19
	fracture/trauma	15
	discitis/spondylodiscitis	14
	cervical stenosis	12
	thoracic	11
	tumor	9
	ambiguous	54
Marital status	married	181
	single	51
	separated/divorced	41
	living with partner	25
	widowed	23
	declined to respond	4
Current work status	employed for wages	151
	retired	79
	disability pension	33
	unemployed	27
	homemaker	22
	vocational training/studies	4
	partial pension	2
	decline to respond	7

Notes. ^a^ Age range [18,1; 87,6]; ^b^ median split

All patients participated voluntarily without payment and signed an informed consent prior to questionnaire administration. Patients completed the questionnaires either at the hospital or in their homes and then returned the questionnaires by mail. The study procedures were approved by the local ethics committee (EK026/15) and conducted according to the Helsinki Declaration.

### Material

The economic-QOL 28-item bank developed by Tulsky and colleagues [8] was translated according to the International Society for Pharmacoeconomics and Outcomes Research (ISPOR) translation guidelines [24]. The translation process included a translation from English into German by three independent translators followed by a discussion of deviations in the translations. The resulting consensus version was backward translated into English by a native English speaker who is fluent in German. Again, deviations between the original version [8] and the back-translation were resolved using group-consensus techniques. The selection of the items was based on the Rasch-analyzed short scale [22] of the original item bank of Tulsky and colleagues [8]. Two items were excluded from the short scale and five items from the broader item bank were retained. This in order to take into account of possible cultural differences and differences in social and health care systems between the USA and Germany (e.g., item 6 “being able to afford the personal care assistance that one needs”), and for being able to distinguish between more fundamental economic needs (e.g., item 4 “I can afford to pay my bills”), and items that relate to items expressing more personal wealth (e.g. item 7 “I can afford to travel”). The final item pool consisted of 11 items with a 4-point Likert-scale scoring ranging from “not at all true” [1] to “totally true” [4] with higher scores indicating a higher agreement with the respective aspect of economic QOL.

Patients’ socio-demographic characteristics (age, gender, family status, and employment status) were assessed by patients’ self-reports and disease specific information (e.g., diagnosis, type of treatment) was extracted from medical records. Group differences based on the Rasch-based economic-QOL-score were assessed by one-way Analysis of Variance (ANOVA) and Tukey’s honestly significant difference test in SPSS 22 [25].

### Rasch analysis

As a model of the item response theory, the Rasch model [26] offers an appropriate framework for item bank calibrations because of its properties of assessing group differences in item and scale functioning, calculating sample invariant item and trait estimates, and transforming ordinal raw score into interval measures [26–29]. In Rasch analyses, response patterns are tested against strict standards for constructing invariant measures. Failure to fulfill the model requirements can contribute to a deeper insight into the construct validity of an instrument and its improvement [30].

In the present analysis, fit to the one-parameter Rasch measurement model was investigated, which involves testing, iteratively, a set of assumptions, including stochastic ordering of the items (fit), unidimensionality, local independence, and properties of invariance across groups, i.e., differential item functioning (DIF), [26, 31–33]. The Rasch analysis was performed using the program RUMM 2030 [34] based on the partial credit model [35], which allows for different use of response categories across items [32]. Details on the following steps of analyses can be found elsewhere [32, 36].

To assess the category functioning of each item, the threshold ordering was examined. A threshold is the point between two response categories in which either response is equally probable. Ordering of the threshold, i.e., three thresholds per item in the present study, was checked using the category probability curves. When disordered thresholds occur the adjacent categories can be merged by collapsing the respective categories [32, 36, 37].

Stochastic ordering is evaluated through considering a series of item fit statistics to indicate adequacy of model fit. An overall fit statistic, the item-trait interaction score, was evaluated by using chi-square statistics; a statistically nonsignificant chi-square value (*p*> 0.05; Bonferroni adjusted) indicates model fit. Individual item misfit was determined by item-fit residuals values (residuals outside the range ± 2.5) [32, 36, 38].

If the response to one item depends on the response to another item, local response dependency is present, which inflates reliability and compromises parameter estimation [32, 39–41]. Local dependency was tested by inspecting the residual correlations. Where response dependency was observed (residual correlations of >0.2 above the average residual correlation), items were merged into testlets to absorb the dependency.

A Rasch analysis allows investigation whether subgroups in the sample respond differently to a given item despite identical levels of the underlying construct [32, 33, 42], a phenomenon called DIF. DIF compromises test fairness, causes bias in measurements, and can influence fit to the Rasch model. In RUMM2030 DIF is evaluated using ANOVA: for each item an ANOVA is conducted comparing person parameter estimates across each level of the person factor (e.g. gender) and across different levels of economic-QOL [32, 36, 40]. In the present study, DIF was tested by gender and age (median split).

Unidimensionality of an item set is a basic assumption when the aim is summing the items into a total score. We tested this assumption using Smith’s test of unidimensionality [43] whereby items loading positively and negatively on the first principal component of the residuals are contrasted through a series of independent *t*-tests. The proportion of significant tests should be less than 5% to support unidimensionality. A confidence interval was calculated to show that the lower confidence interval of the observed proportion falls below the recommended 5% level (LB95%CI).

The Person Separation Index (PSI), a reliability index reflecting the internal consistency of the scale, was calculated. A PSI value ≥0.85 demonstrates a good person separation for individual use and a PSI ≥0.70 is regarded as sufficient for group use [32].

## Results

### Results of Rasch analysis

#### Analysis of the basic model

Response category thresholds were ordered for all items and no DIF was observed for age or gender. The PSI was 0.92, indicating that the items worked well to separate the persons. Initial overall fit (chi-square = 115.3, df = 44, *p* < 0.001) and item fit residuals (M = −0.43, SD = 2.34) were poor. Four of the eleven items showed misfit while the remaining seven showed acceptable fit. Local dependency was identified between a number of items and unidimensionality could not be supported indicated by 9.0% significant *t*-tests with a lower band (LB) of the 95%CI of 6.5 (Table 2).

**Table 2:** Overall model fit of the economic-QOL-scale

Model	Chi-square_(df)_	*p* ^a^	Mean item fit residual	Unidimensionality *t*-test		PSI^d^
				test %^b^	LB95%CI^c^	
Original	115.3_(44)_	<0.001	−0.43	9.0	6.5	0.92
Revised	34.8_(20)_	0.021	−0.04	5.6	3.1	0.88

Notes. ^a^ Bonferroni adjusted = 0.01; ^b^ percentage significant tests; ^c^ lower bound of the 95% confidence interval; ^d^ person separation index

#### Analysis of the revised model

Four testlets were created to account for the local dependencies identified in the basic model: item 1 with the items 5 and 7 (t_1_5_7), item 2 and item 3 (t_2_3), and finally item 4 with the items 8, 9, and 10 (t_4_8_9_10). After adjustment for local dependencies, the data fitted the Rasch model (chi-square = 34.8, df = 20, *p* = 0.021; Table 2). Fit residuals for items (M = −0.04, SD = 1.59) and for persons (M = −0.33, SD = 1.01) were acceptable. Item fit residual values ranged from −1.79 to 2.33 indicating good fit. The unidimensionality of the items bank was supported by 5.6% significant different *t*-tests and a LB95%CI of 3.1. The 11 items were free from DIF. The PSI of 0.88 indicates that the economic-QOL-scale can be well applied in diagnostic of individuals.

**Fig. 1:**
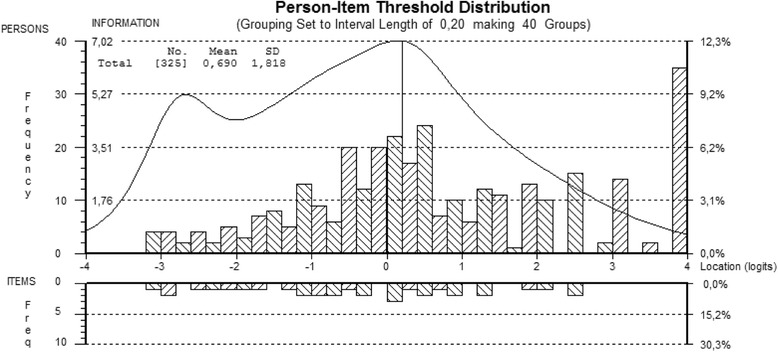
Person-item threshold distribution of the economic-QOL-scale

The category threshold parameters (Fig. 1) covered a range of 5.66 logits (−3.09 to 2.57) with higher values indicating a higher economic-QOL. Figure 1 also visualizes that the scale was reasonably well targeted, covering almost all economic-QOL levels of patients, though about 10% of the sample achieved a maximum score. With a mean person score of 0.69 (SD = 1.82), patients displayed a slightly higher level of economic-QOL than the average difficulty of the scale.

In Table 3 the location (difficulty) of the different testlets, the corresponding standard errors as well as the residuals indicating fit are displayed. The easiest items in the bank belonged to the testlet t_4_8_9_10 that refers to the most basic economic needs (item 4 “I can afford to pay my bills”, item 8 “I have enough income to pay my rent or mortgage”, item 9 “I can afford to feed myself and my family”, and item 10 “I can afford to buy healthy food”) and the most difficult item was item 6 (“I can afford the personal care assistance that I need”).

**Table 3:** Fit of the economic-QOL-scale to the Rasch model

Testlet	Item		Location	SE	Residual
i6			0.79	0.09	2.34
	6)	I can afford the personal care assistance that I need			
t_2_3			0.52	0.06	−1.05
	2)	I have enough income to live the life I want			
	3)	I can afford to live where I want to			
i11			0.24	0.08	0.18
	11)	I have access to extra money in case of an emergency			
t_1_5_7			−0.22	0.05	−1.79
	1)	I can afford to eat out when I want			
	5)	I can afford to participate in the social activities that I want to			
	7)	I can afford to travel			
t_4_8_9_10			−1.32	0.05	0.15
	4)	I can afford to pay my bills			
	8)	I have enough income to pay my rent or mortgage			
	9)	I can afford to feed myself and my family			
	10)	I can afford to buy healthy food			

### Clinical implications

Comparisons based on the Rasch-analyzed economic-QOL-items revealed statistically significant differences in the total economic-QOL-score for separated/divorced versus widowed and married patients as well as for single living versus married patients. Regarding the current work status unemployed patients and patients receiving disability pension showed significant differences in perceived economic QOL scores compared to homemakers, retired patients and those employed for wages (Table 4). There were no statistically significant differences between the remaining socio-demographic or medical variables.

**Table 4:** Differences between groups in perceived economic QOL (raw person estimate)

Socio-demographics	Subgroups compared		Mean difference	*p*-value^b^
	SG1	SG2^a^	(SG2-SG1)	
Age^c^				
	<54.9	≥54.9	0.62	0.002
Marital status				
	Separated/divorced	Widowed	1.42	0.018
	Separated/divorced	Married	1.27	<0.001
	Single	Married	0.79	0.040
Current work status				
	Unemployed	Homemaker	2.39	<0.001
	Unemployed	Retired	2.13	<0.001
	Unemployed	Employed for wages	1.70	<0.001
	Disability pension	Homemaker	2.17	<0.001
	Disability pension	Retired	1.91	<0.001
	Disability pension	Employed for wages	1.47	0.001

Notes. ^a^ SG = subgroup; ^b^ Tukey’s post-hoc honestly significant difference test; ^c^ median split

A transformation table to convert ordinal summative scores into person scores on interval scale estimates is displayed in Table 5. The first column of the table displays the ordinal summative scores, the second column displays the corresponding interval scale latent estimates, which are transformed into a 0–100 interval scale in the third column.

**Table 5:** Transformation table

Ordinal scale score	Interval-scaled person estimate	Transformed interval scale 0–100
11	−3.45	0.00
12	−3.21	3.25
13	−3.02	5.89
14	−2.86	8.04
15	−2.72	9.94
16	−2.56	12.02
17	−2.38	14.52
18	−2.13	17.80
19	−1.87	21.37
20	−1.64	24.53
21	−1.43	27.34
22	−1.24	29.92
23	−1.06	32.32
24	−0.89	34.62
25	−0.72	36.84
26	−0.56	39.01
27	−0.41	41.10
28	−0.26	43.14
29	−0.11	45.10
30	0.03	46.97
31	0.16	48.80
32	0.29	50.59
33	0.43	52.43
34	0.57	54.35
35	0.73	56.42
36	0.90	58.73
37	1.09	61.35
38	1.31	64.34
39	1.56	67.75
40	1.85	71.62
41	2.18	76.12
42	2.59	81.65
43	3.16	89.32
44	3.95	100.00

## Discussion

The present study aimed at calibrating a Rasch-based questionnaire to investigate economic aspects of QOL as perceived by the patients themselves. This scale reflects the subjective appraisal of financial aspects of QOL as opposed to more objective indices of financial status like their monthly salary. It covers a wider range of financial aspects affecting QOL including personal care as well as economic influences related to daily activities and social participation. Economic quality of life therefore represents either a facilitating or debilitating factor concerning activities and participation in social life [8]. By incorporating these facets, the economic-QOL-scale refers to those aspects that patients with SCI consider most relevant [8].

Rasch analyses of the data resulted in a short questionnaire consisting of 11 items that fit the Rasch model when, due to local dependency, 9 items were grouped into three testlets. These testlets represent different types of needs. Whereas the testlet t_4_8_9_10 refers to more basic economic needs, e.g., to pay one’s bills (item 4), the testlets t_1_5_7 and t_2_3 inquire aspects of participation that represent needs of higher order or denote more luxury activities, e.g., to eat out (item 1) or to afford travelling (item 7). Psychometric characteristics were very good in the revised model, indicated by good fit statistics, invariance across group membership, and unidimensionally of the scale. The economic-QOL-scale is invariant for DIF by age and gender. The absence of DIF is important given that income and access to economic resources may vary by age and gender. Users of this item set can be confident that economic-QOL-scores have the same meaning regardless of age or gender [44].

Because the scale was shown to be unidimensional, a total score can be calculated by summarizing the respondents’ answers, with higher scores indicating greater economic QOL. Furthermore, the transformations of the ordinal data to interval scaling can be used to calculate means and SDs in order to compare patients and groups.

Overall, targeting of the scale was good. However, at the positive end of the scale some mistargeting may be present indicated by the absence of item thresholds at higher person locations. To minimize the ceiling effect some more difficult items should be included in future revisions. However, the distinguishing between patients scoring extremely high on economic QOL is probably less important regarding participation than in those patients expressing less satisfaction with their economic situation and facing more financial barriers to participate. Nevertheless, the influence of less basic economic needs influencing perceived economic QOL should also be investigated in order to promote people’s opportunities to live the lives they want to in addition to essential existential aspects.

The scale can be used to improve the recognition and impact of poor economic QOL in patients with SpD and perhaps other disabilities. At the individual level, it may be used to identify persons with a critical level of financial QOL that probably constitutes an important barrier for social and vocational participation as well as a limited access to health care, resulting in increased morbidity and reduced QOL [5, 8, 17]. That is, the identification of critical levels of economic QOL and the accompanying barriers may also provide the opportunity to promote economic facilitators resulting in better QOL, function, and health. Despite the absence of an external validation criterion for defining a cutoff value, a score corresponding to the “mostly not true” rating of the testlet t_4_8_9_10 can be used as indication of a critical value. That is an ordinal scaled raw score of ≤11 on t_4_8_9_10 should rise concerns about potentially problematic low economic QOL that may hamper the person’s participation. A more liberal cutoff value was chosen because failing to accomplish these items in a satisfactory manner; i.e. all four items answered with “mostly true”, would indicate severe constraints in a person’s ordinary daily needs. When this is the case immediate consultations can be conducted by a hospital’s social service, which are anchored in the German health care system.

Regarding the application of the scale for evaluative purposes or at group level, it is possible to identify groups of patients that perceive a low level of economic QOL. For instance, in the present study older persons reported greater economic QOL than younger persons. Unemployed persons and those receiving a disability pension reported lower economic QOL than homemakers, retired patients and those employed for wages.

The economic-QOL-items represent the first Rasch based self-report measure of economic QOL in the German language portraying the patient’s subjective evaluation of their financial status. Furthermore, because of the scale’s closeness to the original items [22], prospective research may validate whether the German and the original scales are comparable respectively results and implications may be cross-culturally compared, at least between the USA and Germany.

The findings of the current study may be limited to in- and out-patients treated for SpD at a hospital setting, which means that the items may not fit the Rasch model when applied in other groups of patients. Therefore, future studies may aim to recalibrate the scale and inspect whether the economic-QOL-scale is transferable to patients with other diseases or patients in different treatment or rehabilitation settings. Another sample related limitation may refer to the application of the scale in other German speaking countries, e.g., Austria and parts of Switzerland and Belgium, with different prevailing legal regulations for health insurance and rehabilitation services than in Germany.

Economic QOL is only one aspect of the ICF-based environmental factors affecting functional health and the different environmental domains are interrelated and may even affect each other [4–8, 16, 19, 45–51]. Poor economic QOL is critical to health related QOL [8]. Therefore it is important to discuss, whether economic QOL has to be seen as a separate and unique concept acting as a facilitating or debilitating factor or whether it should be included as another facet of health related QOL [8]. Prospective studies should evaluate the relationship between economic-QOL with the remaining environmental domains, as well as the predictive validity of the scale with regard to different domains of QOL and functional aspects of health, i.e., daily functioning, participation, or comorbidity. Mapping the processes that impact QOL, function, and health, might also aid to improve health care, rehabilitation, and participation.

## Conclusion

In the study we developed a new Rasch-based German version of the economic-QOL-scale. The instrument was shown to display good psychometric properties and therefore represents a suitable instrument to investigate the influences of economic factors on patients’ QOL at a group and individual level. Because of its brevity, the economic-QOL-scale can be easily applied in research and practice and may therefore be administered quickly in combination with other instruments. The short test duration implies a low test burden for patients and a minimum of time expenditure on the side of clinicians when evaluating the results. The scale provides a first necessary prerequisite for item bank and CAT development and is in its current structure an appropriate instrument to investigate economic aspects of the patients’ QOL.

## Abbreviations

ANOVA: Analysis of Variance
CI: confidence interval
DIF: Differential item functioning
ICD: International Classification of Diseases
ICF: International Classification of Functioning, Disability and Health
ISPOR: International Society for Pharmacoeconomics and Outcomes Research
LB: lower band
M: Mean
PSI: Person Separation Index
QOL: Quality of life
SCI: Spinal cord injury
SD: Standard deviation
SpD: Spinal diseases
TBI: Traumatic brain injury
